# The Hospital. Nursing Section

**Published:** 1905-06-10

**Authors:** 


### The Hospital.

### Nursing Section.

Contributions for this Section of "The Hospital" should be addressed to the Editor, "The Hospital" Nursing Section, 28 & 29 Southampton Street, Strand, London, W.C.

No. 976.—Vol. XXXVIII. SATURDAY, JUNE 10, 1905.

### Notes on News from the Nursing World.

### MRS. WHITELAW REID AND NURSES.

The wife of Mr. Whitelaw Reid, the new American Ambassador to England, who arrived on Saturday to enter upon his duties, in succession to Mr. Choate, takes the utmost interest in the development of the nursing movement. On the other side of the Atlantic Mrs. Whitelaw Reid has manifested this interest in various ways, and she will doubtless extend it to nurses and nursing here.

### RUSSIAN AMATEUR NURSES.

It will be remembered that at the time of the Boer war many ladies, either with relatives amongst the officers or with a desire for adventure, went out to endeavour to get attached to the military nursing staff in South Africa. Evidently history repeats itself, for it is stated that when the Japanese in the recent battle captured the two Russian hospital ships they found a number of Russian ladies on board, amongst them being a niece of Admiral Rojdestvensky, who has applied for permission to nurse her wounded uncle.

### A NURSES' ASSOCIATION FOR NATAL.

The nurses of Natal are to have an association of their own. A movement having this object in view has been on foot for some time, and last month a committee was formed and a secretary appointed. The Mayor of Maritzburg has granted the use of a room in the Town Hall for the meetings of the committee, and about a score of influential personages in the town have been enrolled as honorary members. A considerable number of fully-trained nurses have joined the association, and it is proposed that there shall be a few district nurses attached to it, whose services will be at the disposal of poor people who are unable to pay the full fees. The curious thing is, however, that the latter will be required to pay "about 5s. per day." District nursing as we know it in England has evidently yet to be introduced into Natal.

### THE UP-COUNTRY TOWNS OF CAPE COLONY.

With regard to our warnings to nurses not to go out to South Africa unless they have previously obtained promises of employment, a member of the Army Nursing Service Reserve assures us that from personal knowledge, she can say that though nurses are plentiful in the coast and other large towns, there is great need and almost constant work for the right kind of nurse in the small up-country towns of Cape Colony. But she makes the stipulation that they should not be too young, and should certainly have a little capital. The life, she adds, has many attractions, and the climate is splendid.

### RESIGNATION OF A LONDON MATRON.

After twenty years' service at the Samaritan Hospital, Miss Butler, the matron, has resigned, and is leaving in August. The deep regret felt at her decision is shared by Miss Butler herself, but as she says, "twenty years is a long time to withstand the varied worries and responsibilities which no matron can escape." Miss Butler has spent her entire nursing life at The Samaritan, and has never held an appointment at any other institution. On Wednesday next there is to be a new out-patient department opened at the hospital, when a "farewell" tea will be given to the past and present nurses. Many changes are impending with regard to the staff and the arrangement of some of the wards.

### QUEEN ALEXANDRA'S MILITARY NURSING SERVICE.

We are officially informed that Miss H. B. Derby, Miss L. M. Draper, Miss M. C. Johnston, and Miss M. C. E. Newman have received appointments as staff nurses in Queen Alexandra's Imperial Military Nursing Service; that Miss M. Antrobus, Miss E. F. Armstrong, Miss M. Brown, Miss M. M. A. Copinger, Miss M. Davis, Miss C. D. E. Dunn, Miss E. K. Kaberry, Miss C. G. Lees, Miss A. C. Mowat, Miss S. Richards, and Miss A. A. Steer have been posted as staff nurses to the Military Hospital, Millbank; and that Miss M. L. Kaberry, Miss M. L. Macartney, Miss E. C. Macpherson, and Miss M. B. Williams have been posted as staff nurses to Cambridge Hospital, Aldershot. Miss E. A. Cox, sister, has been posted to the Military Hospital, Millbank, on her return from South Africa, and Miss I. G. Willetts to the Military Hospital, Millbank, from the Connaught Hospital. Miss W. M. Jay, staff nurse, has been ordered from the Cambridge Hospital, Aldershot, to Malta, and Miss M. MacGregor, staff nurse, from the same institution, to Egypt.

### DISMISSAL OF ASYLUM NURSES.

At Stratford Police Court last week three nurses who had been engaged at the West Ham Borough Asylum, Goodmayes, were charged with ill-treating a female patient. The evidence of the chief nurse was to the effect that she saw the patient on the floor, one of the defendants pulling her hair, and the other two holding her hands. A nurse and the medical superintendent said that they saw the patient in the position described by the chief nurse. The defence was that the patient suddenly became violent, struck another patient, tried to bite one of the nurses, and it became necessary to use force to her. The magistrates decided that though there might have been no justification for pulling the patient's hair, it was not intended to hurt her, and they therefore dismissed the case. They were confessedly influenced in their decision by the knowledge that the nurses had already been dismissed from employment at the asylum, and this, they thought, was in any event sufficient punishment. We think so too, especially as the authorities of the asylum gave the three nurses excellent characters, and we hope that the latter will profit by the punishment they brought upon themselves to be careful in the future to refrain from the appearance of evil. A nurse has a perfect right to defend herself when she is attacked by a lunatic, but not to resort to such methods as pulling hair while the patient's hands are held by her colleagues.

### THE EMPLOYMENT OF MALE NURSES CONDEMNED.

An important decision has been arrived at by the Infirmary Visiting Committee of the Fulham Guardians. After giving the system of male nurses for the male isolation block a thorough trial, they report that the result is unsatisfactory, great difficulty having been experienced in obtaining men for the posts, whilst those whose services could be secured have not been properly trained. They recommend, therefore, that, subject to the sanction of the Local Government Board, the staff of male nurses be abolished, and that the guardians return to the old system of female nurses for the ward, the staff to consist of a charge-nurse and two probationers. As to the work in the ward of an objectionable nature, they recommend that it should be assigned to the receiving wardsman with an increase of salary for the duties. The guardians at their last meeting sent back the report to the committee for further consideration, but it is understood that the latter do not intend to depart from the position they have taken up.

### AN UNSATISFACTORY RETURN.

The return issued by Mr. Lowry, Inspector under the Local Government Board for the counties of Northumberland, Durham, the North Riding of Yorkshire, and part of Cumberland, showing the strength of the nursing staff in his district on the 1st of January last, is unsatisfactory. For example, the number of inmates under the care of the medical officers at that date was 3,094, and of nurses on day and night duty 188, an average of 16·86 patients to each nurse, as against an average of 15·94 on the corresponding date of 1904. Moreover, the number of pauper attendants on the sick and also on imbeciles and epileptics in special wards has risen. Examining details, we find that each nurse at Gateshead Union has 28 patients, and at Tynemouth 32. This is in striking contrast with the Unions at South Shields and Newcastle-upon-Tyne, who have 12 and 17 patients to each nurse respectively. It is a matter for regret that progress in some parts of the north-eastern counties should be more than counter-balanced by retrogression in other parts.

### MIDWIFERY IN PLYMOUTH.

Although efforts have been made by the local authority to impress upon those who practised as midwives in Plymouth, before the new Act came into operation, the need of registration, we understand that only 22 out of more than the hundred women hitherto engaged in the work, have applied for the necessary certificate. As the total number of children born in Plymouth last year was 2,880, it is obvious that the supply of qualified midwives in the town is at present far from adequate.

### A NOVEL POINT AT EXETER.

An interesting point arose at the last meeting of the Guardians of St. Thomas, Exeter, on the appointment of the new head nurse. There were only two candidates in attendance, and the choice of the Guardians fell upon Miss Perrett, of Mount Vernon Hospital, Hampstead, a sister of the matron at St. Thomas's Workhouse. The candidature of Miss Perrett suggested the question whether the employment of relatives in the same institution is desirable. A difference of opinion was expressed upon this point, and one of the Guardians, speaking with 30 years of experience, said that twice within that period he had seen matrons at Exeter with sisters as nurses in the workhouse and that the arrangement had worked exceedingly well. In this instance we think that there is not likely to be friction, but so much depends upon the circumstances of the case that no general rule can be laid down with advantage.

### LADY TATE'S GIFT TO BRIXTON.

At the monthly meeting of the Brixton Nursing Association, Miss Reeve, the new superintendent, was introduced to the members, and Lady Tate, as president of the Association, handed into her keeping the "Sir Henry Tate" badge. Subsequently, it was announced by the chairman that Lady Tate had decided to present the Queen's Jubilee Institute with the freehold of a home, the Institute to let it to the Association at a peppercorn rent. The gift includes the furnishing of the house with all necessaries, and it will appropriately be called the "Sir Henry Tate Memorial Home for Nurses."

### PRESENTATION OF MEDALS AT SOUTHAMPTON.

Last week the medals and certificates gained by the nurses of the Southampton Incorporation Infirmary at Shirley Warren, were handed by the chairman of the Board of Guardians to the winners, a number of the Guardians and several invited guests being present. The silver medal was secured by Nurse Firth who obtained 95 marks out of the hundred; the bronze medal by Nurse Rennie who obtained 93 marks, and certificates to Nurses Tayler, Whitren, and Lanham. The Chairman and Dr. Bencraft made brief speeches congratulating all concerned upon the results of the three years of training. Dr. Bencraft said that he felt proud of having such a staff under him as there was at Shirley Warren. He bore testimony to the excellent work done in training by the matron and assistant matron, and to the loyalty of the staff.

### A NURSE INJURED BY THE EARTHQUAKE IN INDIA.

From India we learn that one of the victims of the recent earthquake was Miss Hall, who was at the time nursing a case in the district. She received an injury to her spine and scalp wounds, but was not too badly hurt to be moved to Simla. At the present time she is an inmate of the Walker Hospital, where she is sure to receive every possible attention and care from the lady superintendent and her staff.

### NORTHAMPTON NURSES IN COUNCIL.

A pleasant gathering of nurses took place on Saturday last at the Victoria Home, Northampton, by invitation of Miss Lunn, superintendent of the Northampton Town and County Institution for Trained Nurses. The home is affiliated to Queen Victoria's Jubilee Institute, and four Queen's nurses are supported by the town. Amongst those present were Miss Densham, matron of the General Infirmary; Miss Peterkin, inspector for the Queen's Institute; Miss Kingham, inspector of midwives for the Northamptonshire County Council; Miss Newman, superintendent of the County District Nursing Association, and many others. Miss Amy Hughes gave a sketch of the development of nursing since its organisation by Miss Florence Nightingale, and the foundation of the Nightingale Training School in connection with St. Thomas's Hospital in 1860. She pointed out how the increased education of nurses was necessary to prepare them for the advance in all medical and surgical treatment. Miss Hughes instanced the questions of School Nursing, the working of the Midwives Act, and the inquiry into the need of State Registration as evidence of the wide responsibilities now imposed upon modern nurses, and the need of each one in whatever branch of the work she was employed being adequately prepared, as well as alive to a sense of her personal relations to the profession as a whole.

### £25 FOR A NIGHT NURSE.

At the last meeting of the Strood Guardians it was stated that only one application had been received for the appointment of night nurse, and that the applicant was a widow of 35 with nine months' experience of private nursing. Mrs. Packman, one of the Guardians, said very truly that they could not expect to get a certificated nurse for £25 a year, and this view of the matter was later enforced by Dr. Brown, the medical officer, who mentioned that there was then a case in the House which showed the necessity for both the superintendent nurse and the night nurse being certificated midwives. We strongly advise the Guardians to offer a salary sufficiently high to render it worth while for persons qualified in nursing and midwifery to offer themselves for the vacancy.

### THE ASYLUMS BOARD AND THEIR MATRON.

It will be remembered that last year the Metropolitan Asylums Board appointed Miss Hoatson on trial as head mistress and matron of the training-school section of Darenth Asylum. At the end of 12 months the question of the continuance of the appointment was referred to the Asylums Committee, and they decided to advise against that course, setting forth their reasons at length. At a meeting of the Board the report was discussed, and the Chairman of the Committee, in moving its adoption, said that they had only come to that decision because there was no other alternative. Miss Hoatson, who had given thirty years of faithful, zealous, and good service to the Board, was of opinion that she was capable of doing her work as well in the future as in the past, even if not better, but the Committee considered that she was not so efficient as she had been. They had done all in their power to try and persuade her to fall in with these views, under which she would receive the highest superannuation that the Board could give her. An amendment that the report be referred back for further consideration was proposed, but on being put to the vote, it was lost by 17 votes to 26. A further amendment to defer the matter for a month was also rejected, and the recommendation of the Committee was agreed to.

### NORTH DEVON INFIRMARY LADIES' ASSOCIATION.

It is satisfactory to hear of the rapid progress of the North Devon Infirmary Ladies' Association. In September last year Miss Lillie, matron of the infirmary, convened a meeting of ladies to consider the formation of an association. It was attended by 60 ladies and Lady Susan Fortescue consented to become president. The organisation has now over 90 members and is doing valuable work. The matron acts as honorary secretary. Pound days have been held by ladies in 25 different villages and districts, and 12,000 lb. of groceries, etc., have been sent to the infirmary. A great quantity of clothing has been made for the patients and enough money collected to furnish the infirmary with new bedsteads and mattresses. The patients appreciate the comfort of the spring beds, as they formerly used hard palliasses. Last month an interesting and unique loan exhibition was held in the infirmary at Barnstaple. Curios, valuable paintings, miniatures, china, old silver, and jewellery were sent by the leading families in the district. There was a collection of the best stags' heads secured in the Exmoor country during the past century. Many of the Devon churches sent their old plate; Sir Redvers Buller his sword, and Lord Portsmouth his coronation robes. The exhibition was visited by over 600 people.

### THE PLAISTOW MATERNITY CHARITY.

At the first festival dinner in aid of the Maternity Charity and District Nurses' Home at Plaistow, which was held last week, Colonel Robert Williams, M.P., the chairman, paid a warm tribute to the self-sacrificing work of Sister Katherine, the lady superintendent, and her staff. Having observed that they ought to be supported in every possible way, because they benefit not only the poor who live in Plaistow, but people all over the country, he mentioned that no fewer than 1,750 nurses have been trained at the institution in Howard's Road since its establishment, and that 1,300 nurses who have received training there are now engaged in various parts of the country.

### DRAWING-ROOM MEETING.

A drawing-room meeting will be held at 7 Connaught Place, W., on Wednesday, June 28, at 3.30 p.m., and Lady Hope hopes that a number of nurses will be present. An address will be given by Mr. W. McAdam Eccles, and music and refreshments will be provided. Any nurse desiring to be present should apply for a card of invitation to Miss Hilda Dillon, 47 Oakley Street, Chelsea, S.W.

### The Nursing Outlook.

### THE ESSENTIALS OF A COMMON STANDARD OF NURSE TRAINING.

Mrs. Hunter Robb has done a very real service to the cause of the higher education of nurses by her paper on the affiliation of nurse-training schools. This paper contains many interesting facts concerning the development of nursing matters in the United States during the last fifteen years, and covers most of the subjects agitating the nursing world in these islands to-day. Mrs. Robb lays it down that no effective reform can be hoped for except through the united deliberations and with the common consent of the majority of the training school authorities and those who are immediately connected with the education and training of nurses. She explains that during the last fifteen years individuals and associations in the United States have been busy over improvements in nursing conditions and the education of the nurse. Although fifteen years is not a long time in the history of a great movement of this description, it is long enough, in Mrs. Robb's opinion, to justify the drawing of a certain number of definite conclusions as to the value of the methods so far employed.

The difficulties and serious problems met with have been fully recognised, with the result, that the two great associations of nurses in the United States, *i.e.* the Society of Superintendents and the Associated Alumnæ, now united in one federation, have recognised that if nurses are finally to qualify themselves to enter into the full privileges accorded to members of a profession, three requirements should be presupposed. "First, there must be a definite educational standard; secondly, a proper professional spirit; thirdly, the recognition by the public of this professional standard." It has been the aim of the Society of Superintendents, as it is its primary object, to promote the educational advancement of nurses and the development of a fixed standard of education that should be common to all schools and to all nurses. The Associated Alumnæ, while working for the general uplifting of the nurse and her work, has sought for proper protection by the law, with recognition by the public. In the result certain States have passed Registration Acts, and consequently the lengthening of the term of training from two to three years has been generally adopted in the United States as well as in the United Kingdom.

Mrs. Robb bears testimony to the great development which has taken place in the ground covered by the training of nurses, which now includes many subjects which were overlooked at the outset, as, for instance, the subject of dietetics, of hospital economics, and of household economics in relation to nursing. It is, however, essential, in Mrs. Robb's view, to secure an approximare standard of qualifications for the acceptance or rejection of probationers, and for the dismissal of delinquent students. Steps should be taken to render it impossible, for a woman who has been found unfit as a probationer, or who has been dismissed for reasonable cause at one training-school, to be accepted at another school and ultimately allowed to graduate. Mrs. Robb points out with great force the remarkable fact, that whereas, in almost every instance, some attempt at accepting the whole or some part of some suggested improvement in methods has been made by individual schools, in no single case has the Society of Superintendents ever taken concentrated action, or pledged itself to the general adoption of any one form of improvement, or agreed to accept any standard so far proposed, because it has been felt that such a measure would be impracticable. This result notwithstanding, the Society's efforts have resulted in immense general improvement in nursing in the United States.

At present the nurse-training schools are as far from a general accepted standard of education as they were at the beginning. This leads Mrs. Robb to the conclusion that some new departure, with the view of consolidating the systems of nurse-training, has become, as essential in the United States, as it is undoubtedly essential in the United Kingdom to-day. Mrs. Robb's proposal with the view to bring the nurse-training schools into line in the United States is briefly as follows. She points out that the thing to aim at and desire is to establish a good uniform education for all nurses and for all hospitals. Some such system must be elaborated, and until this is accomplished, our sympathies must lie with the hospitals of limited opportunities. She states, as a matter of fact, that no hospital now exists where such a uniform education can be acquired. In matters of general training the large general hospital offers a wider field for experience tban the smaller hospitals. The special hospitals do not offer scope enough for a full general training, but it is equally true, that the large general hospital has to look elsewhere to supply the training, in certain branches, to round off its course. Hence, the first step to secure is, co-operation between large, small and special hospitals, that the training-schools may be able to offer probationers the necessary practical experience in all branches of nursing. So long as each school remains isolated and indifferent, the results must be unsatisfactory, but the hopeful symptom of the present position is, the existence of a hearty desire almost everywhere for the general betterment of nursing education. Co-operative nursing, *i.e.* a system whereby a central training-school undertakes the training and supply of nurses in all the medical institutions in a given district, has been tried in the United States and has failed. The cost of training is becoming more and more burdensome to the hospitals, and, with the view of relieving them, Mrs. Robb advocates the establishment of Central Institutes in each State, offering a comprehensive theoretical and practical training in general nursing. These Institutes would be independent of any particular hospital, but would be organised and administered by a central committee composed of representatives of the hospitals and schools entering into each State affiliation scheme. Upon some such basis, Mrs. Robb believes, that a solution of the present difficulties can be found, and her instructive paper must have a real interest for nursing authorities in the United Kingdom at the present time.

### The Nursing of Sick Children.

By James Burnet, M.A., M.B., M.R.C.P.Edin., Registrar, Royal Hospital for Sick Children; Clinical Tutor, Extramural Wards, Royal Infirmary; and Physician to the Marshall Street Dispensary, Edinburgh.

### X.—PARALYSED AND SPINAL CASES.

Cases of paralysis and of spinal disease are very frequently met with amongst children. The nursing of these patients forms the principal part of the treatment, and accordingly every nurse must be familiar with the methods to be adopted in the management of these cases. They are too often apt to be regarded by nurses as absolutely hopeless, but not a few of them recover, if not completely, at least partially, provided adequate and competent nursing can be secured.

One of the commonest varieties of case is that known as infantile paralysis. This disease often comes on insidiously with a history of a slight feverish attack. In a day or two one or more limbs, more frequently the lower, is found to be paralysed. After a time the affected limb begins to waste, and the child is quite unable to use it. In such cases more or less permanent paralysis will be almost certain to remain, but a great deal will depend upon the care with which the nurse carries out the instructions we shall now give as to treatment.

There are three important elements in the management of such cases, and these are—(1) warmth, (2) massage, and (3) bathing. These we shall now briefly consider in turn.

1. Warmth is absolutely essential. The affected parts must be well wrapped up. This is best accomplished by swathing the limb in cotton wool, over which a wide stocking should be drawn. This covering should be worn by night as well as during the day. The paralysed limb is usually found to be much colder than its fellow, and it is only by maintaining the temperature of the affected parts that we can hope to achieve any beneficial results, as thereby the circulation is improved, and the chance afforded of restoring the impaired functions of the parts. In addition, care must be taken that the body as a whole is warmly clothed, and the child must always wear woollen combinations both summer and winter.

2. Massage is the part of the treatment which involves most experience on the part of the nurse. To be of any service it must be skilfully applied. Twenty minutes night and morning is not too long a time to employ this form of treatment. Warm olive oil may be used, but the bare hand is preferred by some. The patient should be in bed during the process. The nurse should begin by rubbing the paralysed limb from below upwards. In massaging the right hand should be employed, while with the left the nurse should support the limb that is being rubbed. The muscles should be thoroughly kneaded, using the whole hand and not the finger-tips in carrying out this procedure. In massaging over the bones the nurse should use very little pressure. The front aspect of the limb should first be massaged, and then the back of the limb may be attended to in the same way.

After a time the nurse should cease to rub and knead the muscles, and should gently stroke them with her finger extended and held somewhat loosely. Next the patient should be made to perform movements either alone or aided by the nurse, and in addition he may be trained to resist pressure made by the nurse. Thus she may catch the foot while the patient tries to push her hand away, or she may straighten out the leg while the patient endeavours to bend it at the knee and so on. In this way the muscles are strengthened, and their restoration more certainly assured.

Finally the nurse should rub the whole limb first with her open palm, and then with a rough bath-towel until the skin is red and the limb warm.

3. Bathing has its place in the treatment of paralysis. The limb or limbs may be placed each day in a bath of hot water, care being taken not to scald the child as he may not be able to feel the heat of the water. To the water common salt may be added with advantage. Then the limb may be taken out of the bath and douched with cold water. It must then be thoroughly dried with a bath towel and warm olive oil or lanoline well rubbed into it.

By persevering with these measures, not merely for weeks, but for months, great good may result; and even a little improvement should encourage the nurse to go on with the treatment. This series of instructions is applicable to nearly all cases of paralysis from whatever cause arising.

Spinal caries is a condition due to tuberculous infection of the bones composing the spinal column. It is a serious disease, and one which frequently threatens life. In its treatment absolute rest is the one great essential to success. The spine must be rendered completely immobile. The child must be so fixed in bed that he is unable to raise himself, and the clothing should open at the back so that the garments can be removed without disturbing the patient. In bad cases more will be required, and then resort is usually had to extension of the spine by means of a specially constructed head support which fixes the head and shoulders to the top of the bedstead, while the lower limbs are encased in splints connected with a system of weights and pulleys fastened to the foot of the bed, the latter being slightly raised on wooden blocks.

In advanced cases where the spinal cord has been pressed upon by the inflammatory products formed by the diseased bone we have paralysis, most commonly of the lower limbs; and not infrequently we find that there is both incontinence of urine and of fæces. In such cases bed-sores form very readily, and accordingly the nurse must ever be on the watch for them, endeavouring to prevent their formation by attention to dryness and cleanliness of the skin. She should see especially that there is no undue pressure over bony points, such as the back, heels, hips, and elbows which should rest comfortably in nests formed of cotton wool.

Abscesses sometimes form in connection with cases of spinal caries, and these, of course, necessitate surgical interference for their relief. In no case should they be allowed to burst of themselves, as thereby the patient will run the risk of having the abscess cavity infected with organisms from outside which may produce a severe form of septic poisoning, from which the patient may rapidly die, worn out by the exhaustion occasioned by a continuously high temperature and other hectic phenomena.

Patients suffering from spinal disease should, as far as possible, be treated in the open air. In bright weather the crib should be placed out of doors whenever this can be managed; and, in cases where this is impossible, the windows of the room must be kept widely open. There is no doubt whatever that more rapid progress towards recovery is made in those cases where attention is paid to this important matter. The diet should consist largely of milk, cream, eggs, and raw meat, while cod-liver oil emulsion or virol should be administered two or three times a day.

The class of case we have just been speaking of will often tax the resources of the nurse to the utmost, but if she maintains the patient's spine at rest, feeds him carefully, and supplies him with as much fresh air as possible, while she does not neglect the important matter of bodily cleanliness, the nurse may rest assured that she is doing all that lies in her power to aid the patient's recovery. Time and patience will achieve much, and we have time and again observed successful results follow upon strict attention to the points in treatment which we have tried thus briefly to emphasize.

### The Nurses' Clinic.

### CONSUMPTION. BY A SUPERINTENDENT OF DISTRICT NURSING.

Like most other work, district nursing constantly repeats itself, and the details of management in the following case are applicable to most other cases of consumption.

The patient was a young girl living in a street best known as "Burglars' Rest," but inhabited by some of my most faithful friends and eager pupils. She was just twenty, had lost both father and mother by the same disease, and was supported by her only brother, a labourer in a limekiln. His kindness and goodness to her were extremely touching. On the occasion of my first visit I found her in the kitchen surrounded by the landlady's children, seven in number, and all very young. A good dinner was in course of preparation and a bright fire burning; but it would be difficult to convey any idea of the horrible smell that pervaded the place. The poor girl had a large abscess in her head, which was always discharging, an abscess under the right eye, and a stream of matter continually flowing from her shoulder. It is strange how many people there are to whom one dares not name the word cancer, who are yet convinced that consumption is a romantic and rather pleasant way of dying.

I asked her to come into the front room, and inquired what dressings she had been using. She produced a box of stale zinc ointment and some dirty rags from under the sofa cushion. She was a club patient; but the overworked doctor had long ceased to visit such a hopeless case. As I had, therefore, no directions as to dressings, I followed my usual plan, which is to syringe the wound well with Condy's fluid or boracic lotion, put on a piece of clean rag arranged fourfold and dipped in warm Condy or boracic, cover it with a similar piece of dry rag, and then wool and a bandage. In long chronic cases where wool becomes too serious an expense, flannel that can be washed every day will answer the purpose. I always keep at the Home a large glass syringe (price 2s.). The use of it cleanses the wounds more thoroughly, and exposes the nurse for a shorter time to the risk of infection. I have had a syringe of this kind kept for the use of one patient for two years, and it has never been broken.

My next object was to induce the patient to confine herself to one room, thus strictly limiting the area of infection. The most important point was to get her out of the common living room, and to effect that I went to work gradually but immediately. Experience has taught me that nothing makes so deep an impression upon the minds of the poor as being told the same thing by different people. For this reason, if for no other, the nurse should do her utmost to be on friendly terms with district visitors, Church sisters, and charitable workers of every description. I primed a friend with all the most recently proved facts as to the means by which consumption is spread, and after waiting a few days for the information to reach its destination, I seized an opportunity to warn the landlady of the terrible risks hourly run by her children. "Yes, nurse," she replied, with a worried air, "and that's just what Sister Aleth bin a-saying." To complete my evidence, she fortunately picked up a simple paper on the subject of phthisis written in the parish magazine. The faith of the poor in all that they find "in print" is pathetic and complete, and in about three days I had the girl established in her bedroom and a nice little fire constantly burning.

The next thing to be done was to get the room into good nursing order. The cushions of the chair bedstead were soaked with discharge, and the smell was indescribably foul. I explained to the landlady how injurious such a mattress was, and that in the interests of humanity it must be burnt without further loss of time. I asked her if she had any friend at the stables close by who would give us a large sack of oat chaff. She said she had a brother there, and was sure that he would most willingly give it. I next asked her to buy four yards of Hessian, sew it together and fill it with the chaff. When I returned in the evening the mattress was already made, and the old infected one burnt on a bonfire. The kindness and generosity of many landladies to their lodgers is almost incredible. A lady had recently given me 5s. to be spent for any sufferer as I thought fit, and it seemed that I could not do better than use it in putting the patient's room in proper order, adding to her comfort and making it an object lesson to her numerous friends. As the carpet was very dirty I gave the landlady's eldest child sixpence to take it up and scrub the floor, and then spent threepence on a cake of carpet soap, and asked her to wash the carpet and dry it in the sun. The carpet being very ragged I obtained permission to cut it into strips of a convenient size, which I bound at each end. I then took away all cloths from dressing table, chest of drawers, etc., replacing them by clean newspapers. For the mantelpiece and the little bed-table I provided white paper pinked out at the edges. For the counterpane and short curtains I bought bright washing cretonne at sixpence a yard, and by the time a few flowers had been arranged the room looked fresh and cheerful.

The next most important article to buy was a covered spittoon. I cannot, of course, provide them in all cases, but if there is nothing but an old mug, I never neglect to put carbolic and cold water in the bottom, and to cover it securely with a piece of paper to prevent the possibility of its being touched by house flies. When she became weaker I supplied her

### THE NURSES' CLINIC—*Continued.*

with squares of soft rag, and implored the landlady to burn them immediately after use.

In order to bring what happiness I could into the poor young girl's life, I placed her case before the knowledge of several people well supplied with this world's goods. Only a short time previously an old gentleman had called on me to ask for the names of any chronic sufferers whom he could visit regularly, his one stipulation being that they were to be seriously ill. "I bar grandmothers sitting up in bed eating tinned salmon and toffee!" After receiving full particulars he agreed to visit the girl, and did so until the termination of the case, and sent her flowers and nice little puddings twice a week. "Constancy dwells in realms above," and it is not often that one can find persons who will visit the same invalid week after week, year after year.

A district visitor asked me what she could give the girl. Noticing that, as far as her strength permitted, she was very industrious, I suggested that some coloured wool and knitting-needles would be acceptable, and she was soon happily at work making an elaborate scarf for her brother. She died before it was quite finished, a nurse disinfected and completed it, and it is still treasured by the man as one of the few relics of her young life. After the room was finished I arranged the dressings in a clean wooden box with a lid to it, which from time to time I relined with paper. I dressed the patient's wounds every day, and gave a friendly look round the room, never failing to notice any little change or improvement; but from the day that it was first put in order it was unnecessary for me to do anything with regard to its comfort or cleanliness.

One of the greatest difficulties with regard to phthisis is to induce the friends to have the window open continually, as they are convinced that night air is injurious to the healthy and deadly to the sick. How far the nurse will succeed in making a lasting impression on the friends will of course depend chiefly on their own disposition. In all her suggestions and recommendations and warnings part of Hamlet's advice to the players must be her guide: "Use all gently. . . . Be not too tame neither, but let your own discretion be your tutor."

### Three Months with a Baby in Egypt.

BY AN AMERICAN TRAINED NURSE.

The matron of a hospital and nursing home in Cairo having announced to me that she would want me later for an obstetrical case I paid the customary visit, and found the patient in excellent condition. In January, however, the doctor sent a message to the matron, which read: "Send the nurse with a chart and thermometer." It seems to me that doctors should give some idea as to the nature of their cases at all times when sending notes or telegraphic despatches, this would frequently avoid scandals with regard to the nursing profession. I have seen a three-months' obstetrical nurse sent to a serious medical case through the telegram from the doctor being too concise. In the Cairo case the nurses were thrown into a dilemma, supposing it to be a case of premature labour, and the dressings were in process of sterilising. However, snatching up my handbag, which is always ready for one day's emergency, I hailed an aribeeya (carriage) and drove in haste to find the patient suffering from a severe attack of influenza (temperature 104° F.), which soon yielded to treatment, and in a few days my services were no longer necessary.

### The Arrival of the Baby.

A week later, on February 6th at 5 a.m., I was again called, and on the 8th, with the aid of forceps, a little daughter made her appearance, strongly protesting against being forced into a cold world. She was a healthy, well-nourished child, who quite approved of plenty of flannel beneath her pretty frocks, and of being snugly tucked in her little cot, she was perfectly happy and very sleepy for three days and then her troubles began. Meanwhile the mother's temperature was steadily rising, with no apparent reason. She was treated for sepsis, more for prevention than for cure, but the temperature still kept rising. Tepid sponging was ordered, and on the seventh day a night-nurse was called; on the ninth day rose-coloured spots appeared, making the diagnosis of typhoid positive; on the tenth at 9 a.m. patient's sub-lingual temperature was 106° F., after which it gradually yielded to treatment.

### Feeding the Baby.

And the babe for the first ten days had troubles of her own. The idea that babies could not thrive on Egyptian cow's milk was thoroughly impressed on the mother, the only alternative being either an Austrian or a native woman as wet nurse, which met with the mother's strong disapproval; therefore she determined to nurse it herself, and until typhoid was suspected the doctors were quite glad that she should do so. But it was altogether a hard time for all concerned; the mother's bodily heat was very comforting to the babe, who consequently slept when she should feed, and cried when taken away, causing great anxiety, as the worry was not good for the mother, though she was mentally more satisfied when feeling that she was doing her best for her child. The wee thing, however, got very little nourishment, so I administered sugar-water with a few drops of whisky while waiting developments.

### The Baby Loses Weight.

By the end of a week she had lost three-quarters of a pound; still, the doctor would not allow bottle-feeding, having decided to procure a wet nurse. The Khedive, however, had passed a decree forbidding Austrians to work in Egypt except under certain conditions, and a suitable native could not be found, so it became necessary to instal bottles and sterilising. As baby-nursing was included in my American training I was asked to give up the mother for the child, who was to move at once to the house of her grandparents. The babe was then ten days old and looking very pinched and miserable, so that one could hardly help feeling a weight of responsibility. The child had been nursed by the mother for a week. Would she show symptoms of typhoid? Again, the mother's condition was such that, had evil befallen her babe, the chances of recovery would undoubtedly have been retarded if not rendered altogether improbable; moreover, I was an absolute stranger to the climate and its influence, knew nothing about the quality or property of milk, nor the feeding of cows, and had nobody from whom to gather information. The doctors had evidently come to the conclusion that Egyptian cow's milk was not adapted to the feeding of European babies, having found as results either obstinate constipation or disastrous diarrhœa.

### Egyptian Dairy Supply.

We commenced with milk one ounce to water two ounces, and for the first week I gave whisky, gtt. v., in bottle, three times a day and once at night, because of the babe's weakened condition and the bare possibility that she might develop typhoid. I also took her temperature twice a day. By the end of a week she had gained half a pound, and it was necessary to increase the quality of food to 50 per cent. milk. Owing to constipation, I substituted barley water for plain water, but finding this ineffectual I consulted the doctor, whc agreed to try oatmeal water. This answered admirably, and could be regulated by making it weaker or stronger as indicated, and soon got the intestinal tract into a habit of regularity. It seems to me that the practice of laying down rules for the feeding of babies is a very unwise one, and certainly no rule can be followed in Egypt; the nurse or mother must be guided by the child's progress and general condition. For four weeks it was necessary to increase either the quality or quantity of food. At six weeks old the babe was taking three ounces of milk and one ounce oatmeal-water every two hours per day and twice at night. It seemed a large quantity, so I asked for an analysis of the milk to compare with that of American cow's and mother's milk which are in my possession. Unfortunately the doctor did not remember the constituents, so I had recourse to observation, and concluded that, owing to the immense influx of tourists and visitors the dairy people sold more water than milk. This assumption proved correct; for as the season advanced the milk proved more nourishing, and until three months' old the child took the same quantity and proportions as at six weeks, each week gaining from one-half to three-quarters of a pound in weight. I was interested to learn with regard to Egyptian cows that the instinct for protecting their young is strongly developed; they appear to understand that the semi-barbaric Arab would either neglect to feed the calf, or cause its death through improper feeding, therefore she will not give any milk unless her calf has a drink first; and also the calf must be tied where she can see it during the process of milking. Whether the fact of the calf getting the first milk leaves the dairy a poorer quality I am unable to say. In many cases the farmers mix cow's with camouse milk (a species of buffalo), which is quite white, and of a chalky appearance, but I am told that it is much richer than cow's milk, though not o agreeable to the European palate. Milk must be boiled or sterilised as soon as delivered, otherwise it will not keep. I boiled some on two occasions four hours after delivery, and it turned to curds and whey. Of course, these things affect the feeding of babies, and make the task formidable for the nurse. The advantage of using oatmeal-water till the bowels act with perfect regularity is appreciated when the Khamseens sweep over the country. For a few days before the storm the heat is intense, and houses are kept cool by sedulously closing every window and door and drawing sun-blinds from about 9 A.M. till 7 P.M. daily. Each Khamseen is, however, succeeded by a few deliciously cool and even cold days.

### How Khamseen Weather Affects the Infants.

Khamseen weather covers a space of 50 days, and all around one hears of baby troubles. They get over-heated and are bathed in perspiration or prickly heat; then diarrhœa sets in, probably due to throwing off the flannel garments when the child gets over-heated. Instead of removing flannels and dressing the babe in lovely little lace ruffles, I gave it a small dose of castor oil once a week, using thinner flannel by day and thicker at night, for the nights are always cool in Egypt. In the midst of Khamseen weather another difficulty arises; the child has an apparently unaccountable severe attack of diarrhœa, for which it is difficult to find a reason. But upon inquiring I find that the cows are being fed on burseem—a cheap kind of clover—which is a necessary article of food for animals at certain seasons, but which acts as a purgative if too much is given. The milk naturally reacts upon the babe. During these attacks I gave a dose of castor oil at first, stopped oatmeal-water and substituted lime-water, then from five to ten drops of whisky in bottle four times in twenty-four hours till the termination of the attack—on the principle that no matter what the disease or illness may be, its effect on the force-pump of circulation should be considered. Atmospheric influence, or the changes in cow's food, have the effect of changing the fæces to first grey and then green on exposure, though perfectly normal in appearance and reaction when expelled by the child.

### The Baby's Vaccination.

Vaccine evidently does its work thoroughly in Egypt. Baby was vaccinated when two months old, and had a very sore leg and with it a temperature of 103·5° F. Though she had a few very bad days and nights, she still gained half-a-pound a week. At three months old she had learned to sleep from 7 P.M. to 2.30 A.M. without waking. Owing to the heat we drove at 7.30 A.M. each day; at 9.30 baby had her bath, after which she slept till 12.45. She was the picture of health and of a happy disposition, and was generally pronounced a splendid advertisement for Cairo.

### Incidents in a Nurse's Life.

**Contributions for this column should be addressed to the Editor, and if accepted will be paid for.**

### EXTRACTS FROM THE DIARY OF A PROBATIONER.

*Thursday, Nov. 2nd.*—Arrived at "The General" this afternoon and was taken straight up to the matron. What a charming woman she is! Grey hair, kind eyes, soft voice, tall and stately. I wish I was tall, but at least that pretty clean uniform is bound to make me look fresh and bright. I liked what she said about being punctual, quiet, and very neat and trim.

*Friday, Nov. 3rd.*—What a frightful noise the night nurse made when she called me this morning at 5.30 A.M. I was wide awake, too, and quite ready to get up although it was pitch dark and very cold. Breakfast was rather bewildering

Nurses to right of me,
Nurses to left of me,
Nurses all round me,
—But how they differed.

Anyhow, I shall know the red-haired nurse again, who wears her cap at right angles. I hadn't half finished breakfast when the nurse at the head of the table, whom they call night sister, got up to say Grace; then everyone tramped off to their respective wards, each with a clean apron under her arm. I had reached my ward and was just going to look round when an appallingly severe-looking nurse came up to me and said, "Just help Nurse J. make the beds on the left, clean the taps and all brasses, scrub the bath, then sweep and dust the two side wards, Lawrence and Edward." Before the last word was out of her mouth she was stripping a bed on the other side of the ward. Here was a day's work, the housemaid at home would have been insulted and—

"Come along, nurse, come along, what are you waiting for?" I heard Nurse J. calling.

They make beds at the same rate as they take breakfast, and by the time we had done the twelfth I was breathless and speechless, and yet nurse J. chatted away to the patients as she worked—a smile here, a pat there, and even where the greatest care was necessary she did'nt seem much slower.

Next I started on the brasses—horrid things! They took ages to come bright and made me so hot. Then away to the bath-room; there I was rubbing away at the taps when I felt a hand on my shoulder, and looking up saw matron and sister. Matron said, "Good morning, nurse," but she looked unnaturally solemn and sister's expression was somewhat strained. I was just bending over the washing-bowl once more when I caught sight of myself in the glass. Oh! how dreadful I looked; hot face adorned with some black smears from the globe polish, cap beating a retreat, collar limp, hair escaping on all sides from my cap, and—but enough, I can't stand any more in black and white.

*Saturday, Nov. 4th.*—I was sent to the out-patient department this morning—it seemed as bewildering as the corner by the Mansion House at first—but sister and the porter (whom the patients call "the gentleman in uniform") soon got things into order. The patients fairly streamed in like the rats in "The Pied Piper of Hamelin."

I was just thinking this when sister called, "Look quick, nurse, Dr. A.'s patients wait here, Dr. B.'s there, and so on; go round, please, and see that everyone is in their right place, and answer Mr. E.'s bell, he's the oculist." "Yes, sister." Sister is awfully smart. "If yer please, nurse, where shall I wait?" I turned round to find a woman carrying a baby in what looked like brown paper, three other children were hanging on to her skirt.

"What's the matter?" I asked, "Eyes? poor little thing, here you are, sit down and go in when the doctor rings."

"Thank you kindly, nurse; you 'ave time to speak civil; its mor'n some of 'em 'as. The baby, bless it; been ailing this three week; it ain't me own but my sister 'Arriett's; the poor thing."

"Nurse, nurse, can't you hear that bell?" Sister does make me jump. I fled.

The oculist was standing amidst a little group of students and greeted me with: "Does my bell r ing, nurse?"

"Yes, sir," (I should rather think it did).

"Then please answer it at once another time. Now pack this patient's eye and then she can go."

I was just going to say—"What does pack an eye mean?" when he turned away and began talking to his students again. I was beginning to feel the need for prompt action at any cost, and looking round for inspiration my eyes fell on a grey-haired student standing a little away from the group. So I went up to him and whispered, "Would he tell me how to pack an eye?"

"Just put that pad of gauze over it and then a bandage, but don't bandage it at all tightly."

I carried out these instructions to the letter and went, but hardly had I shut the door when the bell pealed. Ah! never will I ask a student anything again, be he never so grey! When I returned Mr. E.'s face was enough to make a Trojan's heart sink.

"Nurse," pointing a finger of scorn at my handiwork, part of which was on the floor, "did you pack that eye?"

"Yes sir, I—"

"That's enough, nurse, it's disgraceful. Do it again, please."

I wish I had learnt a little bandaging before I came to hospital.

*Sunday, November 5th.*—I did expect a little respite to-day, but just as the chaplain was beginning service, one of the doctors telephoned to say that a case was coming in for immediate operation. So I was sent off for this and that at everyone's beck and call, and finally Sister said I must go with her to the theatre as Nurse J. was off duty. I was rather pleased, thinking I was really going to do some nursing at last. I scrubbed my hands and arms quite merrily until they resembled raw meat juice.

So to the theatre we went. Sister told me all I had to do was to pick up swabs out of a glass jar with the forceps and hand them to the house surgeon.

First only the patient's breathing was heard: then stillness which was oppressive; no one spoke a word. Sister passed everything to the house surgeon without him asking. A few minutes later the surgeon took a small knife (they call it a scalpel, I am told), there was a sharp quick sound, and a hideously scarlet stream of blood followed. The surgeon's head grew misty, some one far away began to speak, the swab at the end of the forceps began to dance and shake, then a voice in my ear said, "Go! go! get out, quick!" I made for the door, managed to get as far as the ward kitchen, and then—no more. When I sat up Nurse was saying, "This will never do; pull yourself together, and when you feel better go and put hot bottles in the operation case's bed."

I felt ready to sob with vexation when the wardmaid came over to me. "Never mind, nurse," she said, "they often gets a turn that way at first; you'll git used to it, never fear. So long as you didn't make no bother in the theatre, Sister won't say nothink. Why, I knows one nurse as come out fairly 'owling the fust time she went in."

"You don't mean it, but she isn't here *now*," I added ruefully.

The wardmaid bent down, and putting her hand to her mouth whispered, "Yes, she is; she's staff-nurse in Wellington."

After that I felt as if I could go and fill those bottles.

*Monday, Nov. 6th.*—I was sent to lecture to-day, but didn't take as many notes as I expected. What a dear old professor—but what words. I must go and buy a nurse's dictionary to-morrow; it strikes me I've a frightful lot to learn yet, and I can't remember a tenth of all I read before I came to hospital. Well, I had better rub my poor blistered feet with some methylated spirit and then to sleep, for I find nights in hospital, to quote Omar, are but

"One moment in annihilation's waste,"

and then the voice of the night nurse proclaims that the stars have set. Well, it hasn't been such a bad day after all——

*Tuesday, Nov. 7th.*—A fearful lot of dressings in the ward to-day. The stock of bandages ran out, so Sister told me to go to the out patient theatre and ask Nurse for some more, and "be quick about it." So off I went, but Nurse was not there. I opened every drawer I could see until I found some, and was back in no time. Half an hour later the out-patient staff came to our ward, and after speaking to Sister came and told me I had left five drawers open. She told me a lot of other things, too. Perhaps I wasn't meant for a nurse, after all. "Life is" (I think) "a blunder and a shame."

*Wednesday, November 8th.*—I shall never forget to-day if I live to be a hundred, there were 10 operations for our ward and the staff-nurse (she's a dear) told me to try and be extra quick, "put on an extra special edition," she said, with a smile.

I tried all I knew and at the end of an hour had broken two medicine bottles and thrown away an important specimen. When I told Sister she gave me a look as if she was counting my vertebræ—then she sent me away to scrub mackintoshes; I suppose she thought it was all I was good for. I was just thinking this when the dear little staff-nurse with the brown eyes put her head round the bathroom door—although Sister had scolded her for the lost specimen she didn't seem to bear me any grudge—"Cheer up, nurse," she said, "take your wiggings with a good grace, but see you don't get two for the same mistake." I certainly shan't if I can help it.

*April 2nd.*—Can it be true I have been here six months to-day? I feel as if I had earned my holiday. I feel ten years older. I wonder if I look it; anyhow, I'm sure the home folks will find me a wiser, if not a sadder, maid.

### Nursing in a Sanatorium.

BY A NURSE.

THERE is no doubt that nursing in a sanatorium is a totally different thing to nursing in a hospital, and that in some ways it calls for a more all-round sort of woman, than is absolutely necessary for the latter. Given the fact that all nurses should be highly-educated women, and so convey an atmosphere of education and refinement to those under their care in hospital, these qualities are positively indispensable in a sanatorium, and the greater the number of social attributes the more valuable will such a nurse be in that particular line.

### AN INDISPENSABLE EQUIPMENT.

Should a nurse proposing to take up this work rely on the fact that she is highly certificated, possessing a splendid surgical or medical training, thoroughly accustomed to ward management, she will find herself indifferently equipped unless there be added to her knowledge and training a social side, a wide humanity and tolerance, and the power in large proportion of adaptability. She must divest herself as much as possible of the professionalism suitable to a hospital but utterly useless in a community whose great idea is to combine the pursuit of health with the most normal conditions attainable, and who prefer to keep as much out of sight as may be the sad fact that they are suffering from a terrible and frequently hopeless disease. Entertainment, therefore, in every shape and form is much sought after, and the good raconteur, the musical proficient, the happy reciter, will find her gifts stand her in lieu of even better and more solid qualities. The call for the latter is, of course, imperative, and if the private nurse with but one patient and an army of friends finds the need of tact an indispensable quality, what is it likely to be in a place containing a family of highly educated people? Moreover, in the majority of cases, it is a long and protracted course of treatment, extending in most cases over six or seven months, and in many instances a much longer period. Also there are the alternations of hope and despair when the patients go away and come back again in order to thoroughly establish the cure. These call for a vast amount of patience, and very clever management to sustain hope in face of so many disappointments. From an eventful point of view, to the ordinary nurse, the work may be termed more or less dull; there is little or no rush, there are no operations, and very few sudden demands for the strung-up energy and brilliant management which form the undercurrent of ward life. A terribly bustling, active, insistently busy woman would be quite out of touch with the lazy, easy, restful life so necessary in what is as its very designation implies, "a rest cure." There must likewise of necessity be a fair amount of what is termed "menial work," that is to say the hated dusting, polishing, cleaning, and incessant tidying which all who have worked their way through "Pro-dom" to the dizzy heights of Sisterdom hope to have escaped for ever.

### THE PLEASANT SIDE.

But having now dwelt somewhat on some of the drawbacks and disagreeables of sanatorium nursing, there is a good deal to be said for its pleasant aspect. Most of these health resorts are placed in delightful surroundings, such as Nordrach, standing in its own charming grounds, and surrounded by miles and miles of forest land; the Arosa Sanatorium in the peaked hills and mountains of the Upper Engadine, with the snow-clad mountains all round it; and in England the sanatoria at Bournemouth, with their beautiful views of sea and wood, or the Kingwood Sanatorium in Oxfordshire, on the top of the Chiltern Hills, typical of perfect English country, with plenty of scope for botanists and naturalists. Moreover, the patients who frequent these sanatoria are usually of an extremely interesting type, embracing people of varied professions, many having broken down through protracted strain of over-work, and being far removed from just the ordinary person with no particular interest attached. For all who suffer from consumption there should be nothing but the deepest compassion, and the utmost longing to be privileged to help them through and nurse them during the long weary period of waiting before a cure can be effected. Nurses in sanatoria should be of a bright and breezy temperament, able to show their undercurrant of intense sympathy by bright cheeriness, and willing to put themselves entirely on one side, to meet the varied mental requirements of the patients. It would be well for them to cultivate a broad-minded interest in all that goes on; they should be fond of reading and able to discuss books and literature. Add to this extremely practical qualities, and a readiness to turn to and do anything and everything without stopping to think whether it is menial or otherwise.

### A HEALTHY LIFE.

The hours off duty are not marked out in the same definite way as hospital hours, but as most of the work lies out in the open, and none of it is very hard work this does not matter. It varies very much according to the season of the year; in winter the cases are usually worse, and such complications as pneumonia, asthma, etc., occur, and the work is more trying, owing to the cold making it difficult to keep the patients warm enough sitting for hours together out of doors. The staff of a sanatorium have in the cold weather a far worse time than the patients running about with blankets and hot-water bottles and food across the snow-bound paths to the shelters dotted all over the grounds. The line between duty and off-duty is a little difficult of demarcation, where part of it may consist of getting up theatricals, concerts, playing mild games of all sorts, and helping in every way to promote the social interests of the place. For nurses who are overworked and tired and permeated with the close air of hospitals, nothing could be better than a course of sanatorium nursing.

### A NEW EXPERIMENT.

At Keith an interesting experiment is being tried of a sort of community open-air treatment, there being a Government staff of doctors and nurses who visit patients in their own homes, and endeavour to teach poor people hygienic conditions of life, what to do with the tuberculous sputum, how to disinfect their clothes, etc. In time there is no doubt we shall have large colonising schemes set afoot for communities of tuberculous people, where work may be carried on under ideal conditions, and where gradually, owing to systematised care, the disease will be prevented from spreading, and if confined to certain centres may in time have some chance of being stamped out. It will be in these altruistic consumptive days that the sanatoria nurse will come to the fore, hence those now nursing in sanatoria may be looked upon as pioneers of a great movement.

### "The Hospital" Convalescent Fund.

THE Hon. Secretary begs to acknowledge with grateful thanks the receipt of 3s. from Miss Marsh, and 7s. 6d. from the Travel Correspondent of THE HOSPITAL.

### A Visit to the University Hospital, Pennsylvania.

BY AN ENGLISH NURSE.

One hour in which to visit a large American hospital is a brief time indeed, but I was leaving the city of Philadelphia that day and did not wish to lose the opportunity offered by a graduate of the medical school to conduct me round the building, which is annexed to the Pennsylvania University. The handsome collegiate buildings stand on several acres of well cultivated land, on the outskirts of the old Quaker city of Philadelphia, now no longer the quiet place of residence which it used to be but a busy commercial centre with a million and a half inhabitants. With the medical school of the hospital are associated the names of men famous both in medicine and surgery; in the board-room hangs a large oil-painting of the celebrated Hayes Agnew, demonstrating in the old clinical theatre, with its wooden table and benches, which remains to-day as a testimony to the advance of surgery within the past quarter of a century. The hospital is not of modern construction and cannot boast of architectural beauty, but its interior appointments are all that can be desired. On entering the wards one is impressed with the severity of their aspect, owing to the strict regard for the principles of asepsis and hygiene. The white-tiled walls, white enamelled beds with their white coverlets, the glass tables serving as lockers, and the white chintz-covered screens, combine to suggest the theatre rather than the ward, but the bright American sunshine streaming through the long windows gives such a sense of warmth and cheerfulness as to fully compensate for the absence of the gay-coloured quilts and screens which we consider so essential to the appearance of our wards. One word about the screens: they are excellent in their construction, consisting of a three-fold enamelled framework covered with white chintz, and run noiselessly on rubber-tyred wheels, thus relieving the nurse from the burden of screen lifting. The charge nurse, who would represent the sister of an English hospital ward, is entirely in white; blue dresses are worn by the graduates in their second year, while the check dresses denote a first-year's nurse; and the probationer, who enters for one month's trial, is allowed to follow her own particular choice in the matter. The superintendent —or matron, as we say—is an Englishwoman, who received her training at the Birmingham Infirmary.

We began our tour of the hospital in the basement, where are the kitchens, linen, and storerooms, next visiting the wards. The Gynæcological ward is the most complete of its kind I have seen; attached to it are an ether-room, an examination-room, and a recovery-room. The patients' charts differ from ours, in that the temperature, pulse, and respiration are indicated by lines distinguished from each other by the use of different coloured inks; this is particularly striking on the medical charts, as showing at a glance the intricate connection between the various functions. The orthopædic ward is well adapted to the special needs of its cases; one end opens into a large room built of glass; here many bright-faced little Americans were cheerfully playing, in spite of their iron-cased limbs, and even the bed-ridden children were wheeled out to sunny quarters. "Glass-houses" are attached to every ward, and as the name implies are entirely constructed of glass, heated to a temperature of 65° F., and cosily fitted up with sofas and the indispensable American rockers. There are two well-equipped operating-rooms; the clinical theatre has seating accommodation for 300 students, and adjoining it is a sterilising-room, and ether-rooms both for men and women. Cleanliness and orderliness prevail, and throughout the hospital it is apparent that the best possible work is being accomplished with the best possible means.

### Presentation to Miss Hull.

BY AN OLD GREAT NORTHERN CENTRAL NURSE.

On Saturday afternoon last the Great Northern Central Hospital was the scene of a gathering of present and past workers, including also many friends and well-wishers. The occasion was the presentation of a silver tea service, including a caddy, a brooch and two very substantial cheques as farewell gifts to Miss Hull, from the nursing staff, on her retirement from nursing work, after an unbroken period of 30 years spent in hospital. For 18 of these she served in the capacity of matron of Great Northern Central.

Miss King, matron of Peterborough Infirmary, late assistant-matron at the Great Northern Central, made the presentation on behalf of the nursing staff, in the board-room, where tea was afterwards served, the tables being tastefully adorned with red and white roses. The neat and workmanlike appearance of the sisters and nurses in their respective dark and light blue uniforms, interspersed with the "gayer adornments" of the outside world, lent a charming touch to the otherwise sombre air of a hospital board-room.

Miss Hull, in returning thanks, spoke in terms of high praise of the loyal and sincere help which she had always received from her nursing staff. It had enabled her to maintain her position as matron over the long period of years which had now closed.

The proceedings terminated with many earnest "farewells," coupled with expressions for *bon voyage*—all present hoping that Miss Hull's journey round the world may prove a happy experience and yield a restful holiday.

### Somerset Nurses' Fête.

BY A CORRESPONDENT.

By the kind invitation of Major and Mrs. Barrett, the Triennial Fête for the Nurses of Somerset, was held on May 30th, in the beautiful grounds of Moredon, North Curry.

The host and hostess had evidently spared neither pains nor trouble for the amusement and comfort of their guests. A loan collection of surgical instruments and sick-room appliances was lent by several London firms: Messrs. Bailey, of Oxford Street; Messrs. Down Brothers, of St. Thomas Street; and the Institute of Hygiene, Devonshire Street. The nurses were delighted and took the greatest interest in all the new and up-to-date appliances. Many went away with gifts of books, tins of food, powders, etc., and bought several articles besides. The baby linen and little dresses called forth much comment and approval from both nurses and the many lady visitors who were also present. There is an impression in some circles that this triennial nurses' fête is only for the nurses belonging to the Somerset County Nursing Association. But this is a mistake. Every matron and nurse belonging to the hospitals, infirmaries, and unions, and even private nurses in the county, are invited as well as the "Queen's," the county and the district nurses; and for weeks in advance a small band of devoted ladies are busy working out the trains to and from the various points, and arranging for brakes and motors to convey the visitors. Thanks are due to all these ladies for their admirable arrangements, which tended so much to the general comfort.

There were about 72 matrons and nurses, besides over 70 other guests. After ices and other light refreshments Mrs. Sanders, of Exford, took the chair, opening the proceedings with an appropriate little speech, at the end of which she introduced Miss Burr, the hon. secretary of the League of St. John's House, the speaker of the day, who gave an admirable address on "The Ethics of Nursing." Then followed a vote of thanks to Mrs. Barrett, proposed by Gen. Emerson and seconded by Miss Elinor Smith, the hard-working superintendent of the nurses of Somerset; also a vote of thanks to Miss Burr, proposed by Miss Joseph (one of the hon. secretaries of the Somerset County Nursing Association), and seconded by Nurse Gaitskell. Nurse Barker proposed a vote of thanks to Miss Eden and the ladies who had helped her in an amusing little speech, her vote of thanks being seconded by Nurse Cross.

The concert which followed unfortunately had to be curtailed, owing to the flight of time. Finally, a most amusing little dialogue entitled, "Joint Household," was thoroughly enjoyable. Before their departure the nurses adjourned to a large marquee, where a substantial tea was prepared, sufficient to fortify even those who had a long cross-country journey in front of them before they could reach their home.

### The Nurses' Bookshelf.

AT THE KING'S RIGHT HAND. By MRS. FIELD. (Wells, Darton, and Co. Price 3s. 6d.)

THIS is a very charming historical tale founded on the study of a boy whose ambition was to serve his king. The graphic pictures of the condition of the country in Alfred's time, helped by excellent illustrations, make it a most interesting and instructive book for boys and girls of any age.

THE WHOLE ART OF MAKING TORCHON LACE. (Cockermouth: John Harris and Sons, Ltd., Derwent Mills. Price 1s. 3d.)

THIS is an excellent little book, dealing in the fullest details with the art of making Torchon lace. The cost entailed is very small, and brings this art within the reach of all. With the assistance of Messrs. Harris's admirable hand-book, it is possible for any intelligent person to become proficient in the making of Torchon lace.

A QUIXOTIC WOMAN. By ISOBEL FITZROY. (London: John Murray. 6s.)

THE heroine of "A Quixotic Woman" is the granddaughter of a Scotch peer. She is entirely the victim of circumstances, as adverse as the means are contemptible, by which a weak, worldly mother attempts to retrieve her own broken fortunes by marrying her to a man with money. The story is not a pleasant one, nor is the author experienced or clever enough to make the characters and the sordid details of the greater part of it sufficiently exciting to sustain the reader's interest. The husband leads a double life, and the victim who shares it as his wife is "The Quixotic Woman."

NELSON'S SIXPENNY CLASSICS.

MESSRS. T. NELSON are publishing an edition of well-known and popular works of fiction which is remarkably attractive. The books are quite small but the type is clear and they are neatly and daintily bound in cloth of various colours with gold lettering. The handy little volumes are especially suitable for presentation, and the works already published form a promising nucleus of a most interesting little library, and they include "A Tale of Two Cities," "Tom Browne's Schooldays," and "The Deerslayer," by Cooper. Another volume is Thackeray's "Henry Esmond." It is the largest book of the series so far and is a wonderful production for 6d.

THE APPLE OF EDEN. By E. TEMPLE THURSTON. (Chapman and Hall. Price 6s.)

MR. TEMPLE THURSTON'S book is a powerful study of a man's temptation and his ultimate victory. The characterisation of the principal actors shows much intuitive perception and the pictures of Irish country life are interesting. Mr. Thurston writes with force and treats the difficult question of a celibate priesthood from a human and impartial standpoint. "The Apple of Eden" was only published quite recently and a second edition has been called for, which proves that it is being widely read and appreciated by the large class of readers who value originality in the conception, and ability in the treatment of a delicate and complex question.

CAIRO OF TO-DAY. By E. A. REYNOLDS-BALL, B.A., F.R.G.S. (London: Adam and Charles Black. 1905. Pages 264.)

LIKE all books from Mr. Reynolds-Ball's pen, "Cairo of To-day" is a thoroughly practical and useful guide. The opening chapter, "How to Reach Cairo," is well-arranged, and enables the traveller to see almost at a glance the pros and cons of the various routes to the city of the Caliphs. Those who are delicate and are thinking of Cairo as a winter residence will do well to study the chapter devoted to "Cairo as a Health Resort." There is much valuable and well thought out information contained in it. When the would-be visitor has decided how much he can afford for his board and lodging in this Ville de Luxe he has only to study "Hotels and Hotel Life" in this handy little book, and he will see at once the best and most advantageous way to lay out his money.

A GARDEN OF SPINSTERS. By MRS. LEE HAMILTON (Annie Holdsworth). (Walter Scott Publishing Co. 6s.)

"A GARDEN OF SPINSTERS" is a collection of short stories, possibly reprints from magazines, a mode of reproduction which usually weakens the individual power of each story for the reader. But these charming studies are so quaint and original that they do not suffer by their near environment. They are the romances of more or less attractive women who have been saved by their own common sense or the inconstancy of their lovers from unhappy marriages, and prefer cheerful singleness to the uncertainties of matrimony. Each story has for its title the name of some old-fashioned plant—"Rosemary," "Love-in-a-Mist," "Myrtle," etc. There is both pathos and humour in the telling, and much graceful imagery in the setting of these delightful little idylls. According to one cheerful spinster, "Spinsters are the only philosophers. They expect quails on toast, but in the end they put up with cold mutton. A man is a very poor quail when marriage is the toast you serve him on. Men make very good friends, but poor partners. If I am to be a philosopher I must remain a spinster. . . . After all, philosophy is more satisfying than philanthropy."

### Everybody's Opinion.

[Correspondence on all subjects is invited, but we cannot in any way be responsible for the opinions expressed by our correspondents. No communication can be entertained if the name and address of the correspondent are not given as a guarantee of good faith, but not necessarily for publication. All correspondents should write on one side of the paper only.]

### THE NURSES OF SHOREDITCH INFIRMARY.

"CERTIFICATED" writes: The first final examination for the completion of three years' training was held at the Shoreditch Infirmary in January, 1902, by Mr. Stephen Paget. The recent examination at this infirmary mentioned in your issue of May 27th was, therefore, the fourth, and not the second, as was stated.

### THE SELECT COMMITTEE OF THE HOUSE OF COMMONS ON NURSING.

MISS E. C. SHANNON, R.R.C., matron of the Western Infirmary, Glasgow, writes: I observe in the report which you give in THE HOSPITAL, June 3rd, of my evidence before the Select Committee of the House of Commons on State Registration of Nurses, you represent me as having said that most of the nurses of this hospital were the daughters of farmers and tradespeople, and very few from the "gentle" class. This report is not correct. I was asked from what class the nurses in the Western Infirmary were taken. I replied that they were not from the domestic servant class, chiefly the daughters of professional men and farmers. I was further asked if I had any of the so-called "gentle" class. I replied, "Very few." In view of my answer to the previous question I took the latter question to refer to county families, hence my reply.

CHILDREN'S HOSPITAL IN NEW SOUTH WALES.

"A. A. H." writes: In reply to "K. Ll. B." *re* used postage stamps I should like to state that I have been told that the old stamps are chemically treated and sold again to the public in small shops such as tobacconists in poor neighbourhoods where single stamps are often asked for. A friend wrote to a gentleman twice asking what was done with the used stamps, stating what she had been told, but no reply was ever vouchsafed. This looked suspicious. I have hitherto always collected stamps for a friend for some hospital, but shall now discontinue doing so. I should not like to encourage fraud and it is certain that no one would buy the stamps if they were useless. I am told that they are used for decorating purposes, but have never met with any so utilised. It would be interesting to find out where their final destination is.

A RULE AND ITS CONSEQUENCES.

"One of the Staff" writes: Will you kindly publish the following in the columns of your paper? Owing to the Matron of the Stockton on-Tees and Thornaby District Nursing Association refusing to comply with an order of the Committee to turn off the gas at the meter every night at 10.30 p.m., thereby losing the services of the staff of five nurses, who protested against such a rule, she has been requested to resign, and, in the event of her refusal, they give her one month's notice. She has served the Association faithfully and well for the space of ten years, and is much beloved and respected throughout the town. The gas has not been abused in any way. The gas-bill is 8s. more than the preceding year, but this is fully accounted for by sickness and the advent of an extra nurse. I should like the opinion of yourself and readers as to whether this is a necessary rule in an institution where the Matron, and, when emergency calls, the nurses also, do night-duty. On the nurses' half-days they are not required to return to the home until 10.30 p.m. I may say that the gas-bill for last year was £10 4s. 4d. The staff consists of matron, five nurses, and two servants. This also includes an outside lamp. Do you think that the Matron's action necessitated such extreme treatment on the part of the Committee?

[If a matron cannot conform to the rules of her committee, however unreasonable they may appear to be, she must, we are afraid, put up with the consequences.—Ed. Hospital.]

THE NURSING OF CHRONIC CASES.

"H. S." writes: There have been a great many letters lately on the much-discussed subject "The Registration of Nurses and the Taxation of the Uniform," and all this is very good so far as it goes; but one writer spoke of the untrained nurse being after registration no longer able to attend to chronic cases, and furthermore added that the "Smart Surgical Nurse" would not think it worth her while to attend to chronic cases. Now such a nurse as this "trained and even registered" is not worthy of the name of woman much less of being called a nurse; a chronic case calls forth our skill and sympathy just as much as an operation case or an acute medical one; and to keep our poor chronic ones clean, comfortable, and free from bed sores is, to my mind, as creditable to any nurse as to nurse the most complicated operation case she could have. When we say and hear others say, "Oh, she is only an old chronic, I am sick of her!" do we ever think that we or a dear mother or father may be the same, and then we shall expect tenderness and care? Let us who call ourselves "women," act as such in word and deed and thought, and let us show our best side to the poor chronic ones we have under our care who feel their helplessness, and often their loneliness, by giving them a "word in season," and tending them with a cheerful spirit.

### Appointments.

[No charge is made for announcements under this head, and we are always glad to receive and publish appointments. The information, to insure accuracy, should be sent from the nurses themselves, and we cannot undertake to correct official announcements which may happen to be inaccurate. It is essential that in all cases the school of training should be given.]

Adelaide Hospital, Dublin.—Miss Mabel Bryan has been appointed sister. She was trained at the Royal Infirmary, Liverpool.

County Nurses' Home, Dorchester.—Miss F. A. Kemp has been appointed on the private staff; she was trained at the Hospital, Fishponds, Bristol, where she was afterwards staff nurse. She has since been temporary charge nurse in the same institution; she is registered under the Central Midwives Board.

Inverness District Asylum.—Miss S. E. Armstrong has been appointed charge nurse. She was trained at the Royal Infirmary, Derby, and has since been sister at the Ruchill Fever Hospital, Glasgow, and at the Hertford British Hospital, Paris.

Morningfield Hospital, Aberdeen.—Miss Margaret S. Purves has been appointed sister. She was trained at Burnley Infirmary, and has since been charge nurse at the Parochial Hospital, Dundee. She has also been district nurse at Bolton and Sain, Ross-shire.

Retford Hospital and Dispensary.—Miss E. M. E. Wood has been appointed staff-nurse. She was trained at the Beckett Hospital, Barnsley, Yorkshire, and has since done private nursing at Wakefield.

Royal Victoria Infirmary, Newcastle-on-Tyne.—Miss Jean Elder has been appointed superintendent of the Nurses' Home. She was trained as lady housekeeper at the Edinburgh School of Domestic Economy, had charge of the Scottish Branch of the Queen Victoria's Jubilee Institute for Nurses, was lady superintendent of the kitchen department of the Royal Edinburgh Asylum, and subsequently had charge of the New Nurses' Home at Woodilee Asylum, Lenzie, near Glasgow.

Victoria Hospital, Blackpool.—Miss Bowman has been appointed staff nurse. She was trained at Rochdale Infirmary.

Withington Workhouse Infirmary, Manchester.—Miss Nellie Littlewood has been appointed superintendent nurse. She was trained at the Chorlton Union Hospitals, and she has since been charge nurse.

### To Nurses.

We invite contributions from any of our readers, and shall be glad to pay for "Notes on News from the Nursing World," or for articles describing nursing experiences at home or abroad dealing with any nursing question from an original point of view according to length. The minimum payment is 5s. Contributions on topical subjects are specially welcome. Notices of appointments, letters, entertainments, presentations, and deaths are not paid for, but we are always glad to receive them. All rejected manuscripts are returned in due course, and all payments for manuscripts used are made as early as possible after the beginning of each quarter.

### Novelties for Nurses.

(BY OUR SHOPPING CORRESPONDENT.)

"IVORIZ."

IVORIZ is an efficacious and agreeable tooth-powder, prepared after the directions of a surgeon-dentist. It is sold in convenient little boxes by the Medical Supply Association, Gray's Inn Road.

SCRUBBS' AMMONIA AND SKIN SOAP.

SCRUBBS' ammonia is a household word. Yet there may be some who do not realise what a useful possession it is and for how many purposes it may be utilised. Firstly, in the bath it is most refreshing and wholesome. It is a good renovator of soiled or spotted garments, applied with a piece of flannel, and diluted with water. It is excellent for cleaning brushes and jewellery or silver, and, in fact, for all cleansing purposes. It is almost an essential for the traveller. Messrs. Scrubbs are now introducing another useful toilet commodity. This is their antiseptic skin soap, which they declare to be free of alkaline mixtures and all that can do harm to the skin, and which contains only materials with emollient properties. It is a most pleasant soap to use, well made and delicately perfumed. Messrs. Scrubbs' address is Guildford Street, Lambeth, but their preparations can be obtained everywhere.

PROVISIONS FOR THE HOLIDAYS.

THE West Surrey Dairy Company realise how difficult it frequently is for travellers to secure good milk, and often any milk at all. They are consequently prepared to assist the public to meet the difficulty by supplying excellent and convenient dried milk. This milk is a powder, put up in boxes, which deteriorates very slowly. All that is necessary to secure good milk for any purpose is to add hot water. It is supplied as separated, half, and whole-cream milk, according to the purpose for which it may be required. The West Surrey Dairy also supply the most delicious butter, cream, and cream cheese. The latter is a real cream cheese, not that unpleasant sour and curdy thing that often does duty for the real article. Thus it is seen that, should it not be procurable elsewhere, an order to the West Surrey Dairy Company will secure, when on holidays or at home, all dairy goods of the very best quality, with certainty and regularity of supply. The address of the head office is Guildford.

### Death in Our Ranks.

THE many friends of the late Nurse Annie A. F. Wheeler, writes a correspondent, will regret to hear of her death at the early age of 30 years. She commenced her nursing career at 17 at Coleraine Cottage Hospital, and a year later was appointed nurse at Mercer's Hospital, Dublin. She then resolved to undergo her three years' training, and remained four years at Lambeth Union Infirmary, passing her examinations brilliantly. She next took six months' special training at St. Peter's Hospital, London, engaged in private nursing at Burton-on-Trent, West Norwood, and Shrewsbury, and finally worked on the staff of the Cathedral Nursing Society, Newcastle-on-Tyne, nursing in a large district of five parishes, till her illness, renal tuberculosis, obliged her to resign in December last. Her great suffering never tempted her to complain, and almost her last words were "Even a nurse must go." She was buried on her birthday in St. Jerome Cemetery, Dublin.

### TRAVEL NOTES AND QUERIES.

BY OUR TRAVEL CORRESPONDENT.

CHANGE OF ADDRESS AT BRUGES (C. E. R.). Mrs. Dear, of Bruges, whose address I gave, now lives at Rue Cour de Gand. Many thanks to the kind correspondent who told me of this change.

CONVENT IN BELGIUM (Catholic). Am sending the address by post. Remember it is extremely quiet near to Namur. Go by General Steam Navigation Company to Ostend, second return 9s.; second return to Namur about £1.

ILFRACOMBE IN JUNE (Theodora). "The Clifton" near the Capstone charges from 31s. 6d. per week, and also "Gilberts'" on the Capstone Parade. These are the only two I know of that come near your terms. It is an expensive place.

SWITZERLAND (H.).—How would you like Meiningen and that neighbourhood? From there you can visit the Lake of Lucerne, the Brunig Pass, Brienz, Interlaken and Thun. Unfortunately, you have seen the finest scenery first, and the rest will seem a little tame. If you do not like this idea, Lausanne or its neighbourhood would be a complete change.

JULY IN PARIS (Paris). July is not a nice month in Paris; it is always expensive then, and I do not think you would make the money last. I should advise a week or 10 days at St. Malo, which is surrounded by excursions, and your money will do that. Second class return £2 1s.; go to Mrs. Dyote, Villa Olga, St. Malo; terms very moderate. Write to her at once on a prepaid foreign postal return card.

SWITZERLAND FOR TWO WEEKS (M. F. L.). The Austrian Tyrol is too far off. What do you think of the top of the Brunnig Pass? The little railway runs continual trains up and down, and you are not shut off. You could visit Lucerne, Interlaken and Brienz easily from there, also Meiningen with the Rosenlain glacier. Hôtel Peus, Kurhaus, Brunig, charges from seven francs. The altitude of Brunig is 3,295 feet.

BRUGES OR KNOKKE (Durban Woman). Stay one week at Knokke so as to visit Middleburg, etc. Distance to Bruges about 15 miles. Roads fairly good for cycling, but you will sometimes meet with miles of pitched thoroughfares. Hotel at Knokke, Hôtel de Bruges. Stay the next week with Mrs. Dear, Rue Cour de Gand, Bruges. You can do this for the money by making the journey to Belgium by General Steam Navigation Company's second class return 9s.

SCOTLAND TO BRUGES (White Heather).—Expenses from London only. Steamer from Tower Bridge (G.S.N.Co.) three times a week. Address, for days of sailing, G.S.N.Co., 33 Great Tower Street, E. Second-class return 9s.; return fare from Ostend to Bruges about 3s. Your journey will occupy four days there and back, so that you have only three weeks in Bruges. Tips, washing, and excursions will be perhaps £3. So I think you can manage it well on what you have. Whilst at Bruges go to Courtrai, Oudenard, Ghent, and Ypres. Of course you can travel second in Belgium. Keep your luggage, you will register it at Ostend to Bruges, where you will produce the voucher given to you. It is no trouble. Change your money at Messrs. Cook's, Ludgate Circus, E.C., and not on the boat. You can do quite well without any French.

RULES IN REGARD TO CORRESPONDENCE FOR THIS SECTION.—All questioners must use a pseudonym for publication, but the communication must also bear the writer's own name and address as well, which will be regarded as confidential. All such communications to be addressed "Travel Correspondent, 28 Southampton Street, Strand." No charge will be made for inserting and answering questions in the inquiry column, and all will be answered in rotation as space permits. If an answer by letter is required, a stamped and addressed envelope must be enclosed, together with 2s. 6d., which fee will be devoted to the objects of "The Hospital" Convalescent Fund. Ten days must be allowed before an answer can be published.

### Notes and Queries.

**REGULATIONS.**

**The Editor is always willing to answer in this column, without any fee, all reasonable questions, as soon as possible.**

**But the following rules must be carefully observed.**

1. **Every communication must be accompanied by the name and address of the writer.**
2. **The question must always bear upon nursing, directly or indirectly.**

**If an answer is required by letter a fee of half-a-crown must be enclosed with the note containing the inquiry.**

*Legal Age.*

(80) Will you kindly tell me if it is legal to sign a document under the age of 21?—*Violet.*

As your question is quite outside this column we must refer you to a solicitor.

*Sanatorium*

(81) Will you kindly tell me of a sanatorium where a young woman suffering from slight lung trouble could be received free of charge?—*L. H.*

See the list of sanatoria, published by the National Association for the Prevention of Tuberculosis, obtainable from the Scientific Press, price 7d. with postage.

*General Training.*

(82) Can you tell me if you know of any hospital where a lady, well-educated, of 37, could get a general training as nurse for two or three years. Would she have to pay a premium?—*M. B.*

She could get general training at several hospitals if she will go as a paying probationer. See "The Nursing Profession: How and Where to Train."

*Children's Hospital.*

(83) Will you kindly tell me if the training for probationers at the —— Hospital for Children is a very thorough one, also a recognised training. It is a small hospital and does not seem very widely known?—*L. J.*

The training at the hospital you mention is considered good, but of course it would be wiser for the sake of your future career to become a probationer at a larger hospital.

*Visiting Nurses.*

(84) Can you tell me if the society or association is formed yet for providing "visiting nurses for the middle classes"? If so, kindly give me the address of the secretary. If the association is not yet formed, could you give me the address of some person interested in the subject?—*Matron.*

There is an association in Marylebone. The honorary secretary and honorary treasurer is the Countess Dowager of Desart, 2 Upper Berkeley Street, W.

*Sulphonel.*

(85) Will you kindly tell me what effect upon the system the occasional and habitual use of sulphonal has?—*J. L.*

No one is justified in taking any hypnotic without the order of a medical man, who, of course, is himself responsible for any "effect upon the system."

*Charge.*

(86) Will you kindly tell me a fair price to charge an invalid lady or gentleman for board, lodging, and attendance weekly?—*H.*

If you will write again, giving full particulars as to your qualifications, accommodation, etc., we shall be able to give you some idea of what would be a fair charge.

*Sanitary Inspector.*

(87) Will you kindly give me particulars as to how a nurse could become a sanitary inspector?—*Inquirer.*

Write to the Secretary, The Sanitary Institute, Margaret Street, W.

*Bandaging.*

(88) Why should a limb be bandaged from below upwards? What complications might occur should the bandage be from above downwards?—*Worried.*

A limb is bandaged from below upwards because that is the direction of the flow of blood in the veins.

**Handbooks for Nurses.**

| | Post Free. |
|---|---|
| "A Handbook for Nurses." (Dr. J. K. Watson.) ... | 5s. 4d. |
| "Nurses' Pronouncing Dictionary of Medical Terms." ... | 2s. 0d. |
| "Art of Massage." (Creighton Hale.) ... ... ... | 6s. 0d. |
| "Surgical Bandaging and Dressings." (Johnson Smith.) | 2s. 0d. |
| "Hints on Tropical Fevers." (Sister Pollard.) ... ... | 1s. 8d. |

Of all booksellers or of The Scientific Press, Limited, 28 & 29 Southampton Street, Strand, London, W.C.

### For Reading to the Sick.

"BLESSED ARE THE PEACEMAKERS."

Down the dark Future, through long generations,
The echoing sounds grow fainter, and then cease;
And like a bell, with solemn, sweet vibrations,
I hear once more the Voice of Christ say "Peace."
Peace! and no longer from its brazen portals
The blast of War's great organ shakes the skies!
But beautiful as songs of the immortals,
The holy melodies of love arise!

*Longfellow.*

"Blessed are the Peacemakers, for they shall be called the children of God."

You are to be peacemakers with one another. "The peacemakers with others are not only those who reconcile enemies, but those who, unmindful of wrongs, cultivate peace. That peace only is blessed which is lodged in the heart, and does not consist only in words. And they who love peace, they are the children of peace." You may be able sometimes to reconcile enemies and make peace between brethren, and so to restore the happiness of many. This is a sacred duty when you can do it.

We must remember that inward peace and outward calm are not one and the same. The most perplexed and persecuted soul may yet be in a state of perfect inward peace. But because there are hostile foes within and around us, whose aim is to destroy our peace, every one who in any degree possesses peace must become a peacemaker. If we will respond to the motions of His grace, and abide willingly in His hand as fellow-workers with Him we shall not miss the promised blessing; only let us keep clearly before us the example of our Master.

Peace was brought not by God alone, in His uncreated nature, but by God Incarnate. The gift is conveyed to mankind through Man. This is still God's method of giving. He uses us that, through the grace of Christ, we may make peace in the world. He leads us to contemplate the majesty and the beauty of His Own peace. He makes us, through faith in Jesus Christ, partakers of His peace; and then He sends us forth to cultivate peace in the world.

What is that serenity, that soft, chastened beauty of the saint which you see even in this life? It is the early glow of that which is to be an eternal glory. There are the Martyrs, the Confessors, there every humble, unknown worker, every peace-lover, every peacemaker, crowned with the joy of perfect life—life that is free for the development of all the grand possibilities which God may have set before them. But, surely, they who in this world have made peace will find, not that their work is ended, only exalted, only glorified, only for the first time perfectly realised.—*Rev. T. Brett.*

There are, in this loud stunning tide
Of human care and crime,
With whom the melodies abide
Of th' everlasting clime:
Who carry music in their heart
Through dusky lane and wrangling mart,
And ply their daily task with busier feet
Because their hearts some holy strain repeat.

*Keble.*

